# Evolution of the Pyrethroids Target-Site Resistance Mechanisms in Senegal: Early Stage of the *Vgsc-1014F* and *Vgsc-1014S* Allelic Frequencies Shift

**DOI:** 10.3390/genes12121948

**Published:** 2021-12-03

**Authors:** Moussa Diallo, Majidah Hamid-Adiamoh, Ousmane Sy, Pape Cheikh Sarr, Jarra Manneh, Mamadou Ousmane Ndiath, Oumar Gaye, Ousmane Faye, Lassana Konaté, Abdul Karim Sesay, Benoit Sessinou Assogba, El Hadji Amadou Niang

**Affiliations:** 1 Laboratoire d’Ecologie Vectorielle et Parasitaire, Faculté des Sciences et Technique, Université Cheikh Anta Diop, Dakar-Fann, Dakar B.P. 5005, Senegal; modiallo@mrc.gm (M.D.); syousmane7@gmail.com (O.S.); akhysarr@gmail.com (P.C.S.); jogomaye@yahoo.fr (O.F.); konatela@yahoo.fr (L.K.); 2 Disease Control and Elimination Theme, Medical Research Council Unit, The Gambia at the London School of Hygiene and Tropical Medicine, Banjul P.O. Box 273, The Gambia; Majidah.Hamid-Adiamoh@lshtm.ac.uk (M.H.-A.); Mamadou-Ousmane.Ndiath@lshtm.ac.uk (M.O.N.); 3 Genomics Core Platform, Medical Research Council Unit, The Gambia at the London School of Hygiene and Tropical Medicine, Banjul P.O. Box 273, The Gambia; Jarra.Manneh@lshtm.ac.uk (J.M.); Abdul.Sesay@lshtm.ac.uk (A.K.S.); 4 Laboratoire de Parasitologie Médicale, Pharmacie et d’Odonto-Stomatologie, Faculté de Médecine, Université Cheikh Anta Diop, Dakar-Fann, Dakar B.P. 5005, Senegal; oumar.gaye@ucad.edu.sn

**Keywords:** evolutionary history, insecticide resistance, *Anopheles gambiae s.l.*, *Vgsc-1014*, genetic diversity, Senegal

## Abstract

The evolution and spread of insecticide resistance mechanisms amongst malaria vectors across the sub-Saharan Africa threaten the effectiveness and sustainability of current insecticide-based vector control interventions. However, a successful insecticide resistance management plan relies strongly on evidence of historical and contemporary mechanisms circulating. This study aims to retrospectively determine the evolution and spread of pyrethroid resistance mechanisms among natural *Anopheles gambiae s.l.* populations in Senegal. Samples were randomly drawn from an existing mosquito sample, collected in 2013, 2017, and 2018 from 10 sentinel sites monitored by the Senegalese National Malaria Control Programme (NMCP). Molecular species of *An. gambiae s.l.* and the resistance mutations at the Voltage-gated Sodium Channel 1014 (Vgsc-1014) locus were characterised using PCR-based assays. The genetic diversity of the *Vgsc* gene was further analyzed by sequencing. The overall species composition revealed the predominance of *Anopheles arabiensis* (73.08%) followed by *An. gambiae s.s.* (14.48%), *Anopheles coluzzii* (10.94%) and *Anopheles gambiae*–*coluzii* hybrids (1.48%). Both *Vgsc-1014F* and *Vgsc-1014S* mutations were found in all studied populations with a spatial variation of allele frequencies from 3% to 90%; and 7% to 41%, respectively. The two mutations have been detected since 2013 across all the selected health districts, with *Vgsc-L1014S* frequency increasing over the years while *Vgsc-1014F* decreasing. At species level, the *Vgsc-1014F* and *Vgsc-1014S* alleles were more frequent amongst *An. gambiae s.s.* (70%) and *An. arabiensis* (20%). The *Vgsc* gene was found to be highly diversified with eight different haplotypes shared between *Vgsc-1014F* and *Vgsc-1014S*. The observed co-occurrence of *Vgsc-1014F* and *Vgsc-1014S* mutations suggest that pyrethroid resistance is becoming a widespread phenomenon amongst malaria vector populations, and the NMCP needs to address this issue to sustain the gain made in controlling malaria.

## 1. Introduction

Despite extensive control efforts, including vector control interventions, over the last two decades, malaria remains one of the main global public health problems. The core malaria vector control interventions long-lasting insecticide-treated nets (LLINs) and indoor residual spraying (IRS) relies heavily on the use of four main insecticides classes recommended for use in public health [1]. Of these, the pyrethroids class of insecticides have been, and remain, the cornerstone of malaria prevention in Africa for almost two decades, including in Senegal. Pyrethroids have been recommended by the World Health Organization (WHO) as the most suitable for bed nets impregnation due to their insecticidal and quick knock-down effects on mosquitoes, while being relatively safe for human and other mammals [2]. Currently, pyrethroids are widely used in all types of LLIN and several IRS campaigns as well as in agriculture [3]. Moreover, several studies have demonstrated the key contribution of LLIN in curbing global malaria transmission and its associated mortality over the past two decades [3,4].

Unfortunately, due to their extremely large numbers and short generation time, mosquito populations evolve very rapidly, and resistance to several insecticide classes, including pyrethroids, are selected in a relatively short time, leading to repeated failures of control intervention [5,6,7]. Resistance to pyrethroids is mainly associated with a single nucleotide polymorphism in the target-site within the voltage-gated sodium channel (*Vgsc*) gene. In the main malaria vector, *An. gambiae s.l.*, two amino acid substitutions have been selected at *Vgsc* locus, *Vgsc-1014F* and *Vgsc-1014S,* respectively known as kdr-west and kdr-east, respectively conferring the cross-resistance to the DDT (Dichloro Diphenyl Trichloroethane) and pyrethroids, and initially identified in western-central and central-eastern Africa, respectively [8]. However, more recently, several findings have suggested the range both alleles being not geographically limited; but found co-occurring in several western as well as eastern African countries, including in Mauritania [9], Cameroon [10], Gabon [11], and Tanzania [12]. According to reports from several West African countries, both of these mutations seemed predominant among the natural populations of the West Africans malaria vectors [8,13,14,15].

Understanding the dynamics and driving factors of insecticide resistance alleles can help in improving resistance management strategies and expend the lifespan of the limited available insecticides [16]. Moreover, with widespread insecticide resistance amongst malaria vectors, WHO lunched a Global plan for insecticide resistance management (GPIRM), which emphasizes the need to improve our knowledge on factors underlying resistance mechanisms [17]. However, no attempts have been made, to our knowledge, to better understand the biological and molecular mechanisms underlying the evolutionary dynamic of both mutations amongst the western African malaria vector populations.

Over the past decade, Senegal has undertaken large up-scaling of pyrethroid-based vector control interventions, mainly through universal the coverage of LLIN and targeted IRS using pyrethroids in selected Senegalese health districts between 2007 and 2010 [18]. However, several reports of pyrethroid resistance amongst natural population of malaria vectors [14,19,20,21], could compromise the gain obtained. Moreover, although the co-occurrence of both *Vgsc-1014F* and *Vgsc-1014S* has been extensively reported from several wild populations across diverse ecological zones of the country [14,20,21], their evolutionary history and dynamic in the country remain unclear and under-investigated. Therefore, understanding the distribution and evolutionary processes of both *Vgsc-1014F* and *Vgsc-1014S* mutations amongst the natural *An. gambiae s.l.* populations as well as their historical gene flow in Senegal, will be critical for the NMCP (National Malaria Control Programme) for its decision making regarding the management strategies of pyrethroids resistance and increase the lifespan of the limited core vector control interventions currently available.

In the present study, the pyrethroids/DDT cross-resistance allele frequencies at *Vgsc* locus, their genetic diversity, and evolutionary history in wild populations of *An. gambiae* *s.l.* were investigated from 10 Senegal NMCP sentinel sites in 2013, 2017, and 2018. These selected years corresponded to three important milestones with the shift of insecticides classes used for IRS across the study area.

## 2. Material and Methods

### 2.1. Study Design

This is a retrospective study tracking-back the evolutionary history of the target site DDT/Pyrethroids cross-resistance in natural populations of *Anopheles gambiae s.l.* across several ecological setting of Senegal. A random stratified sample was drawn from an existing collection of *An. gambiae s.l.* to capture as much as possible the spatial and temporal heterogeneities across the study area.

### 2.2. Study Area and Mosquito Collections

**Figure 1:**
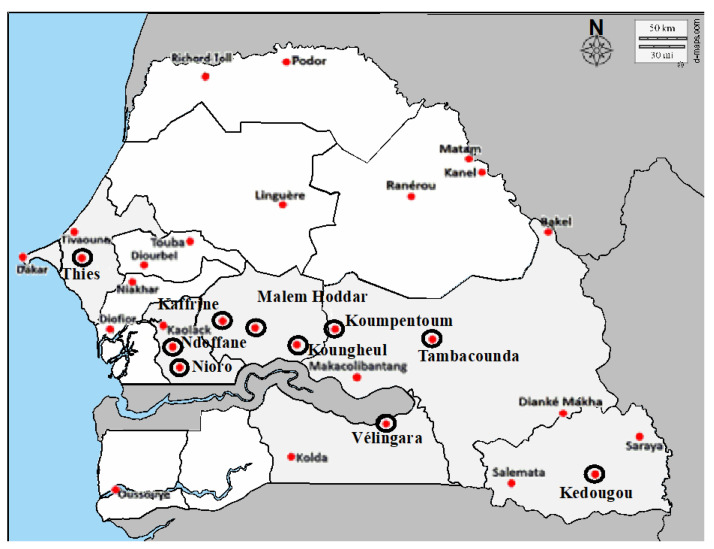
Geographical location of the study Health districts. All the labelled districts here are the NMCP entomological sentinel sites. The selected sites in this study were indicated with black circle.

Historical samples of *An. gambiae s.l.* collected in 2013, 2017, and 2018 were retrieved from ten out of the thirty NMPC’s entomological sentinel health districts (Figure 1). A stratified random sampling approach considering the shifts periods for insecticide classes in IRS was used to select the specimens of *An. gambiae s.l.* stored in the mosquito biobank at the Laboratoire d’Ecologie Vectorielle et Parasitaire (LEVP) which is the entomological reference unit of the NMCP in Senegal. No sample collected during the pyrethroids period was included due to the insufficient samples size and low material remaining after the samples being processed for other purposes.

Entomological surveillance was carried out monthly during the high transmission season from June to November and once or twice (January and/or April) during the non or low transmission season. More detail on the NMCP sentinel sites and entomological surveillance can be found in [18,22].

The ten selected health districts are located in five (5) administrative regions, which in turn belong to our bioclimate zones accounting for almost 90% of the malaria transmission recorded in Senegal: the Sudan-Sahelian (Thies, Nioro, Ndoffane, kaffrine, Malem Hoddar, and Koungheul), the Sudanese zone (Velingara, Koumpentoum, and Tambacounda) and the Sudan-Guinean zone (Kedougou).

Of the ten districts, four (Nioro, Malem Hoddar, Koungheul, Koumpentoum, and Velingara) were previous IRS districts. While the remaining did not receive IRS and were selected as external control health district during entomological survey [18]. From 2007, when IRS started under the US PMI project to 2017, the IRS program in Senegal has gone through several insecticides and classes shift (Pyrethroids: 2007–2011, Carbamate: 2011–2013, Organophosphate: 2013–2017, and No-IRS in 2018) as part of the NMCP’s insecticide resistance management plan. While in the year 2018, no IRS was carried out in any of the selected districts following the decision of the PMI to stop the project in Senegal [18].

### 2.3. An. gambiae s.l. Samples Preparation and Laboratory Processing

Morphologically confirmed 1215 whole specimen of *An. gambiae s.l.* [23], stored individually in silica gel, were selected for subsequent molecular analyses.

The genomic DNA of individual sample was extracted from individual mosquito specimen using automated QIAXtractor Robot (Qiagen, Hilden, Germany). *An. gambiae s.l.* species were identified by species-specific genotyping PCR as previously described by Scott et al. [24] and Fanello Santolamazza and Della Torre [25] at Molecular Diagnostic Laboratories of Medical Research Council, Unit The Gambia at London School of Hygiene and Tropical Medicine.

All the 1215 *An. gambiae s.l.* samples were screened for presence of the two knockdown resistance (kdr) mutations at the 1014 amino acid locus as described previously [26].

### 2.4. Partial Sequencing of Voltage-Gated Sodium Channel Gene Encompassing Resistant Mutations

To infer the polymorphisms of the *Vgsc-1014F* and *Vgsc-1014S* mutations, their origin and evolutionary history, the *Vgsc* gene containing the 1014 mutation flanked by exons 20 and 21 were partially sequenced from 46 individuals (23 *An. arabiensis*, 17 *An. coluzzii,* and 6 *An. gambiae s.s.*) as described [27] at Genomics Core Platform of MRCG at LSHTM in The Gambia.

The samples were amplified using primers (kdr-CL-F: 5′-AAATGTCTCGCCC- AAATCAG-3′) and (kdr-CL-R: 5′-GCACCTGCAAAACAATGTCA-3′). The PCR mixture contained 1x PCR Buffer (New England Biolabs, London, UK), 0.2 mM of each dNTPs (Quiagen, Hilden, Germany), 1 U Taq DNA polymerase (New England Biolabs), and 0.25 mM of each primer, in a total volume of 50 µL. The cycling conditions were as follows: 5 min at 94 °C denaturation step; 35 cycles of 30 s at 94 °C, 30 s at 50 °C, and 60 s at 72 °C; and final extension for 10 min at 72 °C.

Ampure xp beads (Beckman Coulter A63881) were used to purify the amplified product. In detail, 0.9 × of beads was added to the PCR product. This was mixed and incubated at room temperature for 5 min before placing on a magnetic rack. The supernatant was discarded, and the bead–amplicon complex was washed with 80% ethanol twice and air dried before it was resuspended in 25 µL of elution buffer (Qiagen 19086). The supernatant was transferred to a new PCR plate and quantified with the Qubit™ 3 Fluorometer (Ref Q33216). All products were normalized to 20 ng and 10 ng was used as template (based on Thermofisher recommendations for fragment sizes between 500 b-1 kb) for both the forward and the reverse strand cycle sequencing using the Big Dye Terminator cycle sequencing kit (BTCS) V3.1. The master mix for the cycle sequencing consisted of 8 µL of the BTCS ready mix, 4 µL of primer, and 8 µL of PCR grade water. This was subjected to the following cycling conditions: 96 °C/1 min; then 25 cycles (96 °C/10 s, 50 °C/5 s, 60 °C/4 min). Forward and reverse amplification was conducted separately. PCR products were purified with Agencourt CleanSEQ (Ref A29151) and 85% of ethanol using the magnetic stand-96. DNA was eluted in 40 µL of PCR grade water and 20 µL was transferred to the ABI Seqstudio automatic sequencer (Applied Biosystems) for sequencing.

### 2.5. Data Analyses

The *Vgsc-1014F* and *Vgsc-1014S* alleles frequencies were calculated according to the Hardy–Weinberg expectations, and statistical differences among and between the study populations were examined by ANOVA at the significance level of 5%.

The obtained sequences were corrected using BioEdit v.7.2.1 then aligned using the ClustalW [28]. Estimates of DNA polymorphism, including the number of segregating sites, number of haplotypes, haplotype diversity, and nucleotide diversity, were obtained, then the genetic differentiation and gene flow between populations were inferred using DnaSP v.5.10 [29]. The genealogical relations among haplotypes were estimated by constructing network using the Network software v.10.2.0.0 [30] and the maximum likelihood phylogenetic tree was constructed using the MEGA v.7.0 program version [31] with the obtained clean sequences aligned with reference sequences (accession numbers AY615612, EU078898, and EU078896) retrieved from GenBank.

## 3. Results

### 3.1. Molecular Identification of An. gambiae Complex

**Figure 2:**
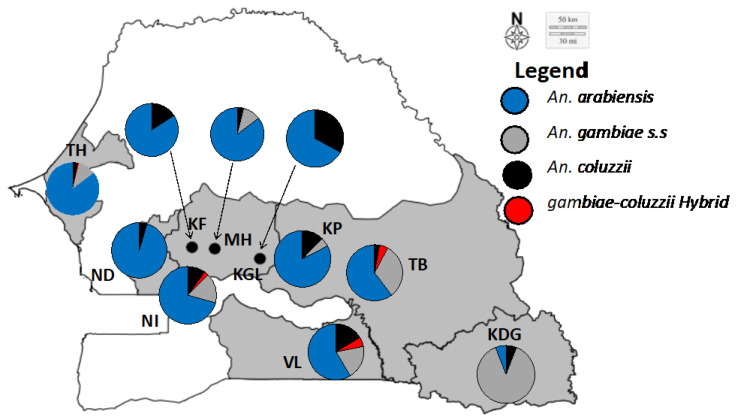
*Anopheles gambiae s.l.* species compositions across the study sites. KF: Kaffrine; KDG: Kédougou; KP: Koumpentoum; KGL: Koungheul; MH: Malem hoddar; ND: Ndoffane; NI: Nioro; TB: Tambacounda; TH: Thiés; VL: Vélingara.

Of the 1215 *An. gambiae s.l*., identified at species level by RFLP-PCR, 888 (73.08%) were *Anopheles arabiensis*, 176 (14.48%) *An. gambiae s.s.*, 133 (10.94%) *An. coluzzii*, and 18 (1.48%) *An. coluzii*–*gambiae* hybrids. *An. arabiensis* was the most frequent and widespread species across the study area and the years, excepted in Kédougou in 2017 and 2018, and Tambacounda in 2013, where *An. gambiae* *s.s.* was the most predominant species, even though being found in sympatry with *An. arabiensis* and *An. coluzzii* (see Figure 2).

### 3.2. Detection of Vgsc-1014F and Vgsc-1014S Mutations

Both the *Vgsc-1014F* and *Vgsc-1014S* alleles were found in the study populations from all the ten health districts.

The *Vgsc-1014F* was recorded in all in the *An. arabiensis* population with an allelic frequency varying between 0.01 and 0.41. It was found in only nine health districts for the *An. coluzzii* population (0.04–0.64), seven districts for *An. gambiae s.s.* (0.11–1), and in two districts for the *gambiae*–*coluzzii* hybrids (0.64–1, see Table 1). The mean of frequencies of *Vgsc-1014F* mutation within sites was 0.10 ± 0.013 for *An. arabiensis,* 0.19 ± 0.03 for *An. coluzzii*, 0.68 ± 0.15 for *An. gambiae s.s.,* and 0.82 ± 0.06 for *gambiae*–*coluzzii* hybrids. The recorded means varied significantly between species (one-way Anova F = 11.61, *p* < 0.001, Table 2). The mean of frequencies of the *Vgsc-1014F* mutation was 0.07 in Kaffrine, 0.45 in Kedougou, 0.27 in Koumpentoum, 0.04 in Kounghuel, 0.39 in Malem hoddar, in 0.04 Ndoffane, 0.06 in Nioro, 0.56 in Tamba, 0.13 in Thies, and 0.65 in Velingara.

On the other hand, the *Vgsc-1014S* mutation was recorded in only nine districts for *An. arabiensis* (allelic frequency: 0.11–0.41, Table 1), three districts for both *An. coluzzii* (0.03–0.30) and *An. gambiae s.s.* (0.05–0.47), and in one site for the *gambiae*–*coluzzii* hybrids (0.07). The means of frequencies of the *Vgsc-1014S* mutation within the districts was not significantly different between species, with 0.18±0.008 for *An. arabiensis,* 0.14 ± 0.04 for *An. gambiae,* 0.09 ± 0.02 for *An. coluzzii,* and 0.07 for *gambiae*–*coluzzii* hybrids (one-way ANOVA, F = 0.52, *p* = 0.67, Table 2). The mean of frequencies of the mutation was 0.08 in Koumpentoum, 0.05 in Koungheul, 0.06 in Malem hoddar, 0.06 in Nioro, 0.04 in Velingara, 0.09 in Kaffrine, 0.03 in Tamba, 0.29 in Thies, and 0.11 in Ndoffane.

Both mutations were found co-occurring in several *An. gambiae s.l.* specimens and populations all over the study period. The frequency of the *Vgsc-1014F* decreased between 2013 and 2018 from 0.46 ± 0.11 in 2013, to 0.19 ± 0.03 in 2017, then 0.22 ± 0.07 in 2018; while in the meantime, the *Vgsc-1014S* mutation frequency remained relatively constant with 0.35 ± 0.09 in 2013, 0.66 ± 0.01 in 2017, and 0.64 ± 0.001 in 2018 (see Table 3). No significant difference was recorded between the allelic frequencies of both mutations over the investigation years (see Table 2).

### 3.3. Genetic Diversity on the Voltage-Gated Sodium Channel Locus

The partial sequencing of a 461 bp of *Vgsc* gene from 46 samples from the 10 districts over the 3-year group, revealed 8 polymorphic sites with a high haplotype diversity of 0.804 and a nucleotide diversity of 0.0059. The genetic differentiation between the 3-year group was significant (Chi2 = 26.572; *p*-value = 0.0219; df = 14) and gene flow showed strong hybridization between populations (F_ST_ = −0.03318).

At the spatial level, each site constitutes a population and the genetic analysis between ten populations showed similar and high levels of haplotype diversity with no significant genetic differentiation between populations (*X^2^* = 77.226, *p*-value = 0.1; df = 63). The overall estimate of gene flow between the populations was moderate with a F_ST_ of 0.1145.

At the species level, the variation in *An. arabiensis*, *An. coluzzii,* and *An. gambiae s.s.* populations showed similar levels of diversity, with an overall haplotype diversity of 0.639, 0.787, and 0.666, respectively. The gene flow between *An. coluzzii* and *An. gambiae s.s.* showed a high inbreeding between their two populations (F_ST_ = 0.02). However, the F_ST_ variation showed a very important genetic differentiation between *An. arabiensis* and *An. coluzzii* but also between *An. arabiensis* and *An. gambiae* with respective values 0.402 and 0.554.

**Figure 3:**
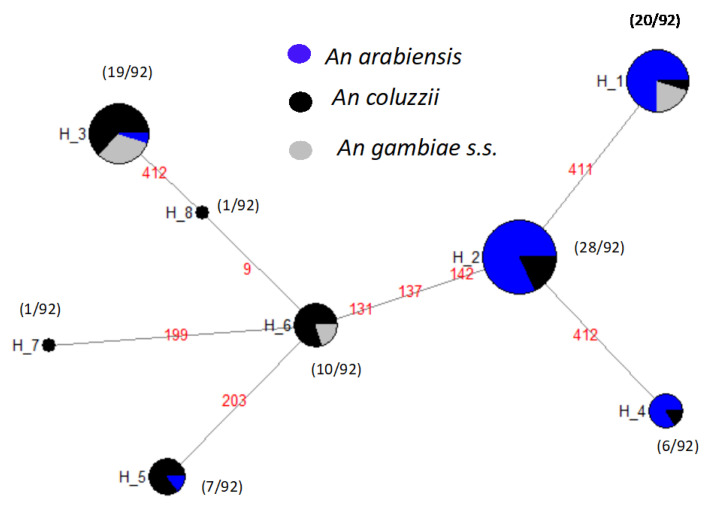
Haplotype network of the *Vgsc-1014* allele in relation to *An. gambiae* *s.l.* species. Each haplotype is represented by a circle with a size proportional to its frequency. The number represent the position of the mutation.

**Figure 4:**
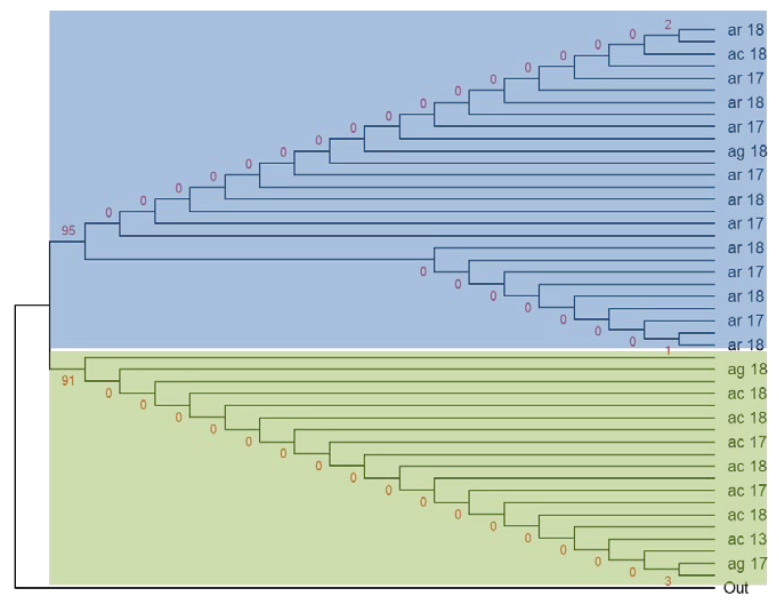
Evolutionary history of the *Vgsc-1014* allele between species and years using the maximum likelihood method. AR: *An. arabiensis*; AC: *An. coluzzii*; AG: *An. gambiae s.s.*; Out: outgroup reference sequence of Vgsc; red number represent the statistics frequency. 13, 17, 18: Correspond to the study years.

The haplotype network (Figure 3) shows that the ancestral haplotype, H2 (28/92) shared between *An. arabiensis* and *An. coluzzii* was specific for both alleles. H1 (20/92) was specific only to the *1014S kdr* and carried by *An. arabiensis*, *An. coluzzii,* and *An. gambiae s.s*. H3 (19/92) was specific to the *1014F kdr* only and shared by all three species. H4 (6/92) and H5 (7/92) were specific to kdr west and carried by *An. arabiensis* and *An. coluzzii*. H6 (10/92) shared between *An. coluzzii* and *An. gambiae s.s.* belonged to kdr west. The lowest haplotypes H7 (1/92) and H8 (1/92) were specific for the *1014F* resistance allele and belonged to *An. coluzzii* (Figure 3). Moreover, the analysis of the maximum likelihood phylogenetic tree between *An. gambiae s.l.* showed two main clades: the major with the three species, and the second made up only by *An. coluzzii* and *An. gambiae s.s. (*Figure 4). However, in the each of these clades, the most of nodes have poor support, suggesting low differences between the individuals. Consistent with the haplotype network, the *Vgsc* haplotypes H1, H2, and H4 were clustered into the top clade, which comprised mainly *An. arabiensis,* while the second clade contained the haplotypes H3, H5, H6, H7, and H8 (see Figure 3 and Figure 4).

## 4. Discussion

Assessing the dynamic of insecticide resistance in major malaria vectors and its impact on the effectiveness of control tools is essential for implementing appropriate strategies to manage the growing challenge of resistance in malaria vectors. In the present study, the resistant allele frequencies at *Vgsc* locus in ten health districts in 2013, 2017, and 2018 were investigated, and their genetic diversity was analyzed to infer their origin and evolutionary history.

### 4.1. Dynamic of Pyrethroid-Target Site Resistant Alleles within An. gambiae Species in Senegal

Molecular characterization of genetic mutations conferring resistance to target site insecticides revealed the presence of both the *Vgsc-1014F* and *Vgsc-1014S* mutations in natural populations of the *An. gambiae sensus lato*. In this study, distribution, and evolution of *Vgsc-1014F* kdr mutation showed its widespread presence and at higher frequencies in *An. gambiae s.s*. and *gambiae*–*coluzzii hybrids* compared to *An. arabiensis* and *An. coluzzii*. Many studies reported this mutation at high frequency within *An gambiae s.s.* and *An*. *coluzzii* especially in *An*. *gambiae s.s.* populations from the south-eastern part of the country [20,32]. The low frequency of *Vgsc-1014F* observed in *An. arabiensis* is consistent with a previous study from the country [14,33]. The low *Vgsc-1014F* frequencies in *An. arabiensis* suggests that this specie is less exposed to selection pressure of insecticides due to its outside resting behavior by avoiding the contact with IRS insecticides sprayed inside houses.

*Vgsc-1014S* mutation was recorded at highest frequency in *An. Arabiensis* populations in the Dakar urban area [34] and in the central-west [14]. Previous studies have recorded only a few individuals of *An. coluzzii* from the central-west part of the country carrying this mutation [14,20]. However, in *An. gambiae s.s., Vgsc-1014S* was found only in heterozygous form as well as in the southern and south-eastern part of the country [20]. The present study reveals that this mutation has spread across the whole country since 2013, and is now observed at a relatively high frequency in *An. arabiensis* but low in *An. coluzzii*, *An. gambiae s.s.,* and their hybrid. However, in *An. gambiae* s.s. and the hybrid form, only the heterozygous mutations were recorded. The observed frequencies are low compared with previous studies in Burkina Faso [35], and in Gambia [15,36].

The difference in the allelic frequency of *Vgsc-1014F* and *Vgsc-1014S* mutations may be related to their origin or linked to different ecological and/or behavioral characters between species. The occurrence of the *Vgsc-1014F* mutation in *An. coluzzii* has been suggested to have occurred by introgression from *An. gambiae s.s.* and via a de novo mutation event in *An. arabiensis* [37]. However, the origin of the *Vgsc-1014S* mutation in *An. gambiae s.s., An. coluzzii,* and *An. arabiensis* species in West Africa is not so clearly understood. It could have been introduced to Senegal by migration, as previously suggested in Burkina Faso [35]. The presence of the *Vgsc-1014S* mutation in the current study is consistent with several reports from West African countries such as Benin [13], Burkina Faso [38], Côte d’Ivoire [39], Mali [40], and The Gambia [36].

### 4.2. Temporal and Genetic Evolution of Pyrethroid Resistance at Vgsc in Senegal

The substitution in the *Vgsc* gene was initially found in the oldest collection in Senegal [41], being in agreement with this present study. However, the trend here indicates that *Vgsc-1014F* frequency has decreased, likely from 2013 to 2018. Historically, Senegal started implementing IRS in 2007 with the financial and technical support from the U.S. President’s Malaria Initiative. IRS has been conducted with different pyrethroids and formulations between 2007 and 2011. The NMCP shifted to bendiocarb from 2011 to 2014, then to organophosphate between 2014 and 2017 [22]. However, no IRS was conducted in 2018 due to the decision of the PMI to stop the program in Senegal [22]. All these insecticide management strategies may have contributed to the reduction in the pyrethroids selection pressure and thus the frequency of *Vgsc-1014F* mutation in the local vector populations [35]. Moreover, the frequency of the *Vgsc-1014S* mutation is very similar in all three years and appears to be fixed in the population, which could result in a selective mechanism to maintain the balance between the allelic frequencies of the two mutations. The evolution of the two mutation frequencies over the years has been analyzed at a complex level due to limited sample size. With the three species of the complex pooled and analyzed all together, no inter-species difference can be missed. However, with *An. arabiensis* being the dominant and most widespread species across the country, this limit could be relativized.

The study observed that variations in the *Vgsc* gene was high amongst the *An. gambiae s.l.* populations. Vector species from different ecological zones showed different haplotypes for this locus, confirming the existence of moderate gene flow barriers between species. These results corroborate with previous studies [42]. Furthermore, of the eight haplotypes recorded, H1, H2, and H3 were found to be the most widespread as previously reported by Pinto et al. when investigating the multiple origins of knockdown resistance mutations in *An. gambiae* sampled across several sites from 15 West African countries [27]. H1 was *Vgsc-1014S*-specific and predominated in *An. arabiensis* in all study districts except Kedougou and Malem Hoddar. H2 was detected in both *Vgsc-1014F* and *Vgsc-1014S* mutations and common in *An. arabiensis,* in all study districts. H3 was more prominent in *An. coluzzii* and present only in the *Vgsc-1014F* mutation. The high prevalence of H3 in *An. coluzzii* contrasted with previous results [20,43].The observed haplotype diversity at the Vgsc gene could be explained by the influence of different evolutionary forces such as gene flow and selection pressure. These forces may lead to changes in the genetic structure of vector populations associated with environmental variation.

## 5. Conclusions

The present study documented spatial and temporal evolution of pyrethroids kdr target-site resistance mechanisms across hotspot areas in Senegal. Both the *Vgsc-1014F* and *Vgsc-1014S* mutations were found in three *An. gambiae s.l.* species, with a spatial variation in allelic frequencies. The presence of the two mutations was detected since 2013 across the country. At species level, the *Vgsc-1014F* mutation was more frequent in *An. gambiae s.s.*, whereas *Vgsc-1014S* more prevalent in *An. arabiensis*.

The observed co-occurrence of *Vgsc-1014F* and *Vgsc-1014S* mutations confirms that pyrethroid resistance is a widespread phenomenon among malaria vector populations. Thus, the NMCP needs to urgently address these findings to maintain the current gain and ensure the success of future control interventions. Other control strategies such as larval source management and targeting of mosquito reproductive inhibitory genes could complement current tools to control pyrethroid resistant vectors.

## Figures and Tables

**Table 1:** *Vgsc*-*1014* alleles frequencies in natural *An. gambiae s.l.* population by study locality.

	Locality	N	Genotypes						Allelic Frequencies		
			FF	FS	LF	LL	LS	SS	*Vgsc-1014F*	*Vgsc-1014S*	*Vgsc-1014L*
AR	Kaffrine	90	2	1	5	58	22	2	0.06	0.15	0.79
	Kedougou	4	0	0	1	3	0	0	0.13	0.00	0.88
	Koumpentoum	95	0	3	5	67	17	3	0.04	0.14	0.82
	Koungheul	86	0	0	2	65	19	0	0.01	0.11	0.88
	Malem_Hodar	121	0	4	9	76	23	9	0.05	0.19	0.76
	Ndoffane	106	1	4	12	48	36	5	0.08	0.24	0.68
	Nioro	92	0	2	8	50	30	2	0.05	0.20	0.75
	Tambacounda	85	2	0	4	58	20	1	0.05	0.13	0.82
	Thies	134	11	16	11	20	59	17	0.18	0.41	0.41
	Velingara	75	28	2	3	28	14	0	0.41	0.11	0.49
	**Total**	**888**	**44**	**32**	**60**	**473**	**240**	**39**	**0.10**	**0.20**	**0.70**
AG	Kedougou	60	60	0	0	0	0	0	1.00	0.00	0.00
	Koumpentoum	6	4	0	0	1	1	0	0.67	0.08	0.25
	Malem_Hodar	1	1	0	0	0	0	0	1.00	0.00	0.00
	Nioro	22	0	0	5	16	0	1	0.11	0.05	0.84
	Tambacounda	45	41	0	3	1	0	0	0.94	0.00	0.06
	Thies	17	0	3	2	2	7	3	0.15	0.47	0.38
	Velingara	25	23	0	1	1	0	0	0.94	0.00	0.06
	**Total**	**176**	**129**	**3**	**11**	**21**	**8**	**4**	**0.77**	**0.05**	**0.17**
AC	Kaffrine	17	0	0	3	13	1	0	0.09	0.03	0.88
	Kedougou	4	0	0	2	2	0	0	0.25	0.00	0.75
	Koumpentoum	14	0	0	1	12	1	0	0.04	0.04	0.93
	Koungheul	42	1	0	4	37	0	0	0.07	0.00	0.93
	Malem_Hodar	8	0	0	2	6	0	0	0.13	0.00	0.88
	Ndoffane	5	0	0	0	5	0	0	0.00	0.00	1.00
	Nioro	13	0	0	2	11	0	0	0.08	0.00	0.92
	Tambacounda	4	1	0	0	3	0	0	0.25	0.00	0.75
	Thies	5	1	0	0	1	3	0	0.20	0.30	0.50
	Velingara	21	11	0	5	5	0	0	0.64	0.00	0.36
	**Total**	**133**	**14**	**0**	**19**	**95**	**5**	**0**	**0.18**	**0.02**	**0.80**
AG-AC	Nioro	3	0	0	0	3	0	0	0.00	0.00	1.00
	Tambacounda	7	7	0	0	0	0	0	1.00	0.00	0.00
	Thies	1	0	0	0	1	0	0	0.00	0.00	1.00
	Velingara	7	4	0	1	1	1	0	0.64	0.07	0.29
	**Total**	**18**	**11**	**0**	**1**	**5**	**1**	**0**	**0.64**	**0.03**	**0.33**

LL: Leucine homozygous (*Vgsc-L1014L*)/Wide Type (WT); FF: Phenylalanine homozygous (*Vgsc-F1014F*); SS: Serine homozygous (*Vgsc-S1014S*); LF: Leucine-Phenylalanine heterozygous (*Vgsc-L1014F*); LS: Leucine-Serine heterozygous (*Vgsc-L1014S*); FS: Phenylalanine-Serine heterozygous (*Vgsc-F1014S*); AR: *An. arabiensis;* AG: *An. gambiae s.s*.; AC: *An. coluzzii*; AG-AC: *gambiae*–*coluzzii* hybrids; N: Number of specimens tested per health district. The alleles frequencies for each species are indicated in bold.

**Table 2:** Variance of the means *Vgsc-1014F* and *Vgsc-1014S* frequencies between study years.

Genotypes	*Vgsc-1014F*					*Vgsc-1014S*				
Source of Variations	SS	*df*	*F*	*p*-Value	*F crit*	SS	*df*	*F*	*p*-Value	*F Crit*
Between groups	0.0847	2	0.7135	0.5020	3.4928	0.0115	2	0.3306	0.7220	3.4434
Within groups	1.1865	20				0.3819	22			

**Table 3:** *Vgsc*-*1014* alleles frequencies in natural *An. gambiae s.l.* population by study year.

	Locality	N	Genotypes						Allelic Frequencies		
			FF	FS	LF	LL	LS	SS	*Vgsc-1014F*	*Vgsc-1014S*	*Vgsc-1014L*
2013	Tambacounda	47	30	0	2	14	1	0	0.66	0.01	0.33
	Thies	32	2	5	2	5	12	6	0.17	0.45	0.38
	**Total**	**79**	**32**	**5**	**4**	**19**	**13**	**6**	**0.46**	**0.19**	**0.35**
2017	Kaffrine	42	2	0	2	23	13	2	0.07	0.20	0.73
	Koumpentoum	51	1	2	4	34	7	3	0.08	0.15	0.77
	Koungheul	64	1	0	3	49	11	0	0.04	0.09	0.88
	Malem_Hodar	66	0	3	7	41	11	4	0.08	0.17	0.76
	Ndoffane	61	1	3	8	25	20	4	0.11	0.25	0.64
	Nioro	66	0	1	10	45	9	1	0.08	0.09	0.83
	Tambacounda	64	18	0	2	29	14	1	0.30	0.13	0.58
	Thies	63	7	6	6	8	30	6	0.21	0.38	0.41
	Velingara	61	31	1	7	16	6	0	0.57	0.06	0.37
	**Total**	**538**	**61**	**16**	**49**	**270**	**121**	**21**	**0.17**	**0.17**	**0.66**
2018	Kaffrine	65	0	1	6	48	10	0	0.05	0.08	0.86
	Kedougou	68	60	0	3	5	0	0	0.89	0.00	0.11
	Koumpentoum	64	3	1	3	45	12	0	0.08	0.10	0.82
	Koungheul	64	0	0	3	53	8	0	0.02	0.06	0.91
	Malem_Hodar	64	1	1	4	41	12	5	0.05	0.18	0.76
	Ndoffane	50	0	1	4	28	16	1	0.05	0.19	0.77
	Nioro	64	0	1	5	35	21	2	0.05	0.20	0.75
	Tambacounda	30	3	0	3	19	5	0	0.15	0.08	0.77
	Thies	62	3	8	5	11	27	8	0.15	0.41	0.44
	Velingara	67	35	1	3	19	9	0	0.55	0.07	0.37
	**Total**	**598**	**105**	**14**	**39**	**304**	**120**	**16**	**0.22**	**0.14**	**0.64**

LL: Leucine homozygous (*Vgsc-L1014L*)/Wide Type (WT); FF: Phenylalanine homozygous (*Vgsc-F1014F*); SS: Serine homozygous (*Vgsc-S1014S*); LF: Leucine-Phenylalanine heterozygous (*Vgsc-L1014F*); LS, Leucine-Serine heterozygous (*Vgsc-L1014S*); FS: Phenylalanine-Serine heterozygous (*Vgsc-F1014S*); N: Number of specimens tested per health district. The alleles frequencies for each year are indicated in bold.

## Data Availability

Data supporting the conclusions of this article are included within the article. Raw data will be made available upon request to the corresponding author.
